# Integrative analysis of the gut microbiome and metabolome in a rat model with stress induced irritable bowel syndrome

**DOI:** 10.1038/s41598-021-97083-z

**Published:** 2021-09-02

**Authors:** Yue Hu, Fang Chen, Haiyong Ye, Bin Lu

**Affiliations:** 1 Department of Gastroenterology, First Affiliated Hospital of Zhejiang Chinese Medical University, 54 Youdian Road, Hangzhou, 310006 Zhejiang China; 2 Department of Gastroenterology, Zhejiang Integrated Traditional and Western Medicine Hospital, Hangzhou, Zhejiang China; 3 Zhejiang Chinese Medical University, Hangzhou, Zhejiang China

**Keywords:** Irritable bowel syndrome, Microbiome

## Abstract

Stress is one of the major causes of irritable bowel syndrome (IBS), which is well-known for perturbing the microbiome and exacerbating IBS-associated symptoms. However, changes in the gut microbiome and metabolome in response to colorectal distention (CRD), combined with restraint stress (RS) administration, remains unclear. In this study, CRD and RS stress were used to construct an IBS rat model. The 16S rRNA gene sequencing was used to characterize the microbiota in ileocecal contents. UHPLC-QTOF-MS/MS assay was used to characterize the metabolome of gut microbiota. As a result, **s**ignificant gut microbial dysbiosis was observed in stress-induced IBS rats, with the obvious enrichment of three and depletion of 11 bacterial taxa in IBS rats, when compared with those in the control group (q < 0.05). Meanwhile, distinct changes in the fecal metabolic phenotype of stress-induced IBS rats were also found, including five increased and 19 decreased metabolites. Furthermore, phenylalanine, tyrosine and tryptophan biosynthesis were the main metabolic pathways induced by IBS stress. Moreover, the altered gut microbiota had a strong correlation with the changes in metabolism of stress-induced IBS rats. Prevotella bacteria are correlated with the metabolism of 1-Naphthol and Arg.Thr. In conclusion, the gut microbiome, metabolome and their interaction were altered. This may be critical for the development of stress-induced IBS.

## Introduction

Irritable bowel syndrome (IBS) is a highly prevalent and multifactorial functional gastrointestinal disorder, and is characterized by altered bowel habits that lower a patient’s quality of life^1^. IBS has multiple triggers^2,3^, such as psychological^4^ and genetic factors^5^, bacterial gastroenteritis^6^, and gut microbiota, which has been studied intensively in recent years^7^. Researchers have found that stress and stress-related factors could lead to IBS-like symptoms, such as visceral hypersensitivity, dysmotility and altered colonic permeability^8,9^. The stress-induced rat model has been well used for the basic study of IBS and IBS-related disease.

It is presently well-accepted that the gut microbiota is critical in physiological function, regulating host immunity and metabolism, which is also affected by environmental factors inside and outside of the host^10,11^. The gut microbiota and metabolites secreted by the microbiota are important components of the intestinal mucosal micro-ecosystem. A growing number of studies have indicated that changes in the intestinal mucosal micro-ecosystem are correlated with the development of IBS^12–14^. However, the altered gut microbiota pattern in IBS conditions remains unclear. In addition, microbial- and host-derived metabolites can regulate many life activities, including cell signal release, energy transfer, intercellular communication, and so on^11^. Therefore, the functional change of the gut microbiota may be reflected in the intestinal metabolome. Thus, identifying the gut microbiota and metabolites composition, as well as the interrelation between these, may be helpful in understanding the mechanism of IBS.

It was hypothesized that the stress-induced microbial component and function changes create a metabolic environment that favors IBS development. Colorectal distention (CRD) and restraint stress (RS) are usually used for constructing the IBS model^15,16^. The present study employed the IBS model, 16S rRNA gene sequencing, and UHPLC-QTOF-MS/MS-based metabolomics approaches to explore the alterations and correlations of the gut microbiota and metabolic phenotype of the IBS rat model through stress treatment.

## Materials and methods

### Animals

A total of 20 male Sprague-Dawley rats, which were 6–8 weeks old and weighed approximately 240–260 g, were purchased from the Animal Laboratory of Zhejiang Chinese Medical University. These rats were raised in cages under a temperature-controlled room with a light cycle of 12 h of light and 12 h of darkness, and received ad libitum access to food and water. This animal study was approved by the Animal Experimentation Ethics Committee of Zhejiang Chinese Medical University, with approval number ZSLL-2016-145; ZSLL-2016-129 (Animal ethics approved by Mr. Wang Dejun in September 2016). All experiments were performed in accordance with ARRIVE guidelines. All procedures were performed in accordance with relevant guidelines’ in the manuscript.

### IBS model establishment and sample collection

**Figure 1:**
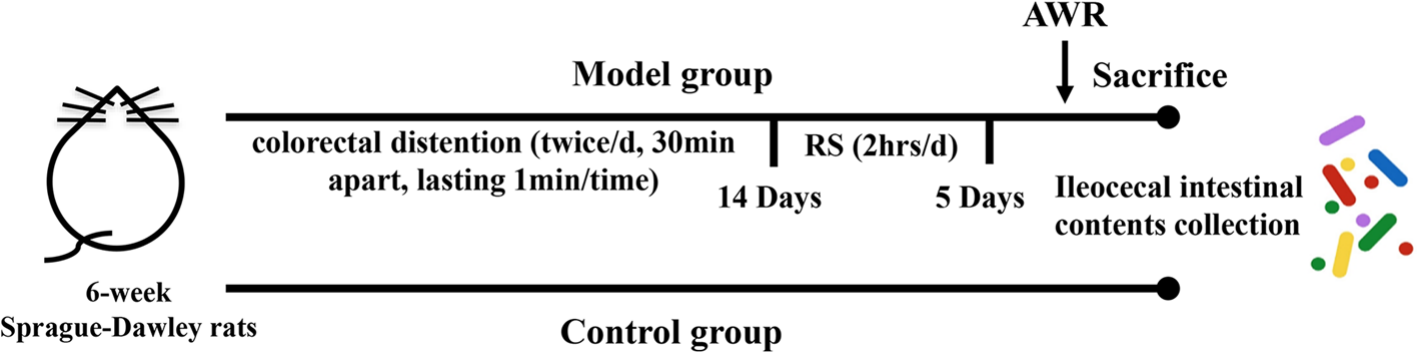
Design of the IBS model using male Sprague-Dawley rats (control group: n = 10; IBS group: n = 10).

In the present study, rats were randomly divided into two groups: control group (n = 10) and IBS group (n = 10). The IBS model was established, as previously described^17^. In brief, IBS rats were subjected to 14 days of colorectal distention, and subsequently treated with restraint stress for five days. These experiments were performed between 10:00 a.m. and 12:00 p.m. each day. Rats in control group did not receive the stress treatment (Fig. 1). After the 19-day consecutive treatment, the IBS and control group rats were sacrificed under systemic anesthesia using pentobarbital sodium. Then, the ileocecal contents were immediately collected and fresh frozen at − 80 °C for further microbiome and metabolome analysis.

#### IBS model evaluation

Visceral hypersensitivity was measured by grading the behavioral response of rats^18^, and this was evaluated by a blind observer through visual observation of the abdominal withdrawal reflex (AWR)^19,20^. The AWR for each rat was evaluated for three times by two independent operators. The mean AWR value was taken to eradicate any discrepancies, and used for the following statistical analysis.

### Microbiome analysis

The genomic DNA of the gut microbiota was extracted from the ileocecus content using a QIAamp DNA Stool Mini Kit (Qiagen, Hilden, Germany), according to manufacturer’s instructions. The quality and quantity of DNA were evaluated by Nanodrop 2000 ultraviolet spectrophotometry. The bacterial DNA was amplified using universal primers 343f, 5′-TACGGRAGGCAGCAG-3′ and 798r, 5′-AGGGTATCTAATCCT-3′ by targeting for the V3-V4 hypervariable region of the bacterial 16S rRNA gene^21^. The reverse primer contained a sample barcode, and both primers contained the Illumina sequencing adapter. Then, the PCR products were purified and quantified, and were used for sequencing on an IlluminaMiseq PE300 platform (Illumina, San Diego, USA) using a 300-bp paired-end sequencing protocol. Quality filtering was performed for the raw paired-end reads using the Trimmomatic software (version v.0.38)^22^. The paired-end read was assembled using the FLASH software (version v.1.2.8)^23^.

The optimized sequences were assigned to operational taxonomic units (OTUs) using Uparse (version v.10.0.240), according to a 97% identity cut-off^24^. The most abundant sequence of each OTU was selected as the representative sequence, subjected to the RDP classifier for taxonomical assignment, and compared with the Greengenes taxa (version 13.8), the NCBI 16S Microbial database (ftp://ftp.ncbi.nlm.nih.gov/blast/db/16SMicrobial.tar.gz), and SILVA databases (version 132)^25^. OTUs cluster, α-diversity and β-diversity calculation, and rarefaction analysis were performed using the MOTHUR program (version v.1.35.1). The relative abundances of the gut microbiota in the control and IBS groups were expressed as mean ± standard deviation (SD), and analyzed by one-way analysis of variance (ANOVA) with Fisher’s least significant difference (LSD) post hoc test. Then, the microbiota and metabolites were evaluated for the predictive accuracy of IBS by receiver operating characteristic (ROC) curve analysis.

The functional capabilities of the gut microbiome were predicted by PICRUSt^26^. The high-level phenotypes that were present in the microbiome were predicted using BugBase, which is a microbiome analysis tool for determining the relative abundance difference of seven microbial communities. The differences between each group were analyzed using Wilcoxon rank-sum test. A P-value of < 0.05 was considered statistically significant.

### Metabolomic (UHPLC-QTOF-MS/MS) analysis

The ileocecus contents sample preparation was processed according to the report of Lv et al.^27^, with minor modifications. Then, 0.2 g of the ileocecus contents sample was diluted using 1 ml of methanol, mixed for 15 s, and allowed to stand for five minutes. Afterwards, the mixtures were shaken upside-down for 15 min, and centrifuged at 12,000 rpm for 15 min. The resulting supernatant was filtered and used for the metabolic profiling analysis.

The UHPLC system (1290, Agilent Technologies) was used for the chromatographic separations. A Triple TOF mass spectrometer (MS) was used to acquire the MS/MS spectra during the LC/MS experiment^28^.

The raw data obtained from the UHPLC-QTOF-MS/MS runs were analyzed using XCMS4dda v1.41.0 and XCMS4lipid v1.41.0. Then, the processed data set was exported and analyzed by SIMCA-P (v14.1, Sweden). The normalized data (peak area normalization) was used to perform the OPLS-DA analysis (the data scaling was performed using Pareto scaling [PAR]; and this was validated by sevenfold cross-validation and response permutation testing [RPT]), which was taken to characterize the metabolic perturbation between the control and IBS groups. Student's *t* test was used to identify metabolites that significantly differed between the two groups. The metabolic pathways of metabolites correlated to the Kyoto Encyclopedia of Genes and Genomes (KEGG) (http://www.genome.jp/kegg/) database were analyzed using MetaboAnalyst (http://www.metaboanalyst.ca).

### Statistics analysis

The statistical comparisons were analyzed using SPSS 22.0 statistical software (Chicago, USA). The experimental data were presented as mean ± standard error of the mean (SEM). A *P*-value of < 0.05 was considered statistically significant. The correlation analysis between two differentially significant variables (16S rRNA and metabolites) was carried out using the Spearman rank correlation test (Spearman’s rank correlation test, p < 0.05, correlation coefficient > 0.6 or < − 0.6).

### Ethics approval

This article was approved by the Animal Experimentation Ethics Committee of Zhejiang Chinese Medical University (approval number ZSLL-2016-145; ZSLL-2016-129). (Animal ethics approved by Mr. Wang Dejun in September 2016).

### Consent to participate

We declare consent to participate.

### Consent for publication

We declare consent for publication.

## Results

### Assessment of the IBS model

**Figure 2:**
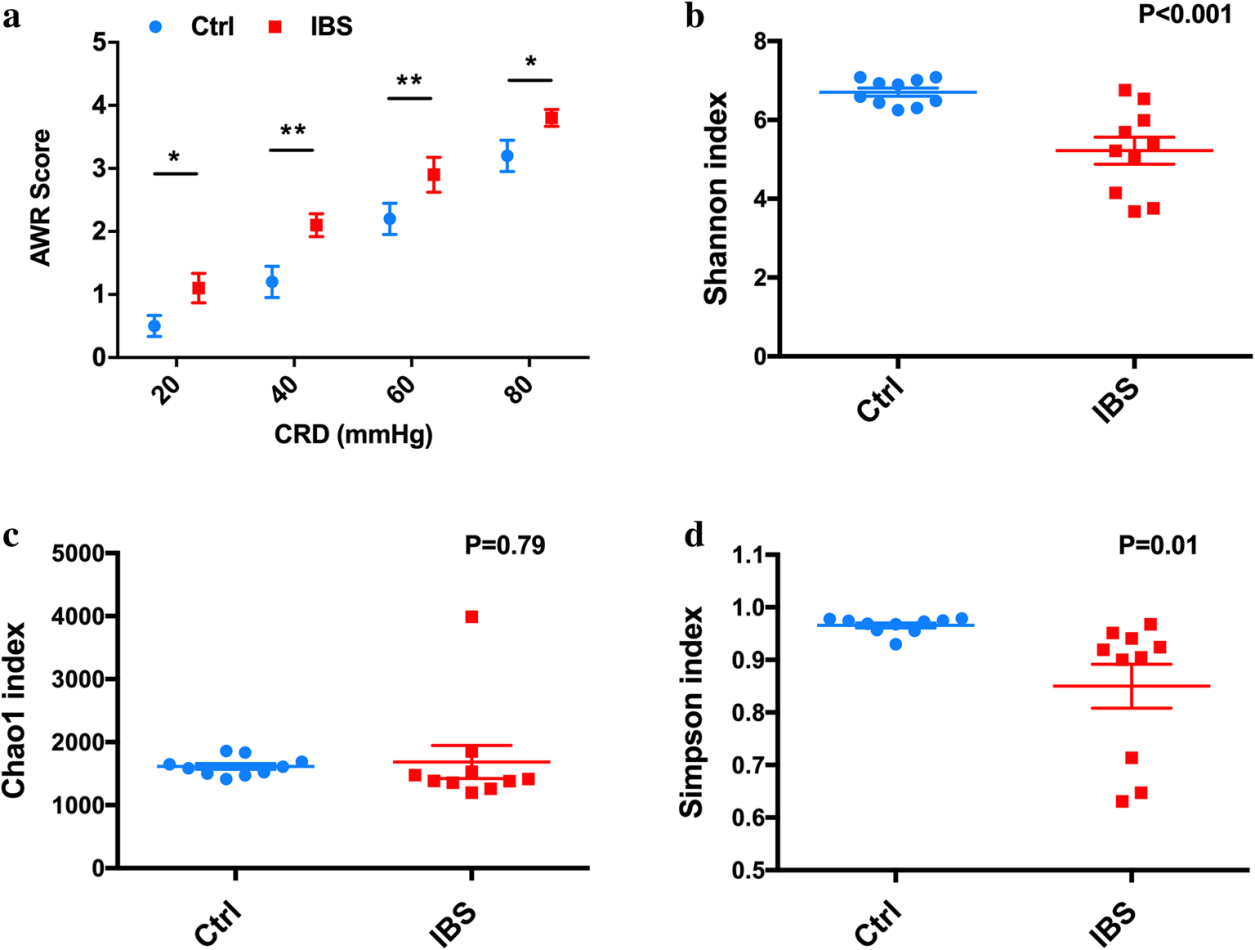
Assessment of the IBS model and the diversity and richness of the gut microbiome between control and stress-induced IBS rats. (**a**) The abdominal withdrawal reflex (AWR) scores in response to stress were evaluated in the control and IBS groups, *P < 0.05; **P < 0.01; (**b**) The microbial diversity characterized by the Shannon index between the control and IBS groups, P < 0.001; (**c**) The microbial diversity characterized by the Simpson index between the control and IBS groups, P = 0.01; (**d**) The microbial richness estimated by the Chao1 index between the control and IBS groups, P = 0.79.

The rats in IBS group were subjected to 14 days of colorectal distention and 5 days of restraint stress. After the 19-day stress treatment, the model rats appeared listless and irritable. In addition, shrugged hair and reduced activity were observed. Furthermore, the stools were thin and soft, accompanied by perianal fecal residue. The mean AWR scores of IBS rats were higher, when compared to those of the control rats, at colorectal distention pressures of 20, 40, 60 and 80 mmHg (20 mmHg, *P* = 0.015; 40 mmHg, *P* = 0.009; 60 mmHg, *P* = 0.000; 80 mmHg, *P* = 0.048; Fig. 2a).

### The gut microbiome altered in the IBS model

A total of 732,648 reads (with 99.5% distributed in 400–500 bp length) were obtained from 20 samples, and were identified to 6538 OTUs at 97% similarity level. The declines in the species diversity were observed, as characterized by the Shannon Wiener index and Simpson index (Fig. 2b,c). However, no statistically significant difference was observed in mean community richness, as estimated by the Chao1 index (Fig. 2d) between the control and IBS groups. The principal coordinate analysis (PCoA) revealed that the diversity of microbiota did not significantly differ between the control and IBS samples (Supplemental material 1).

**Figure 3:**
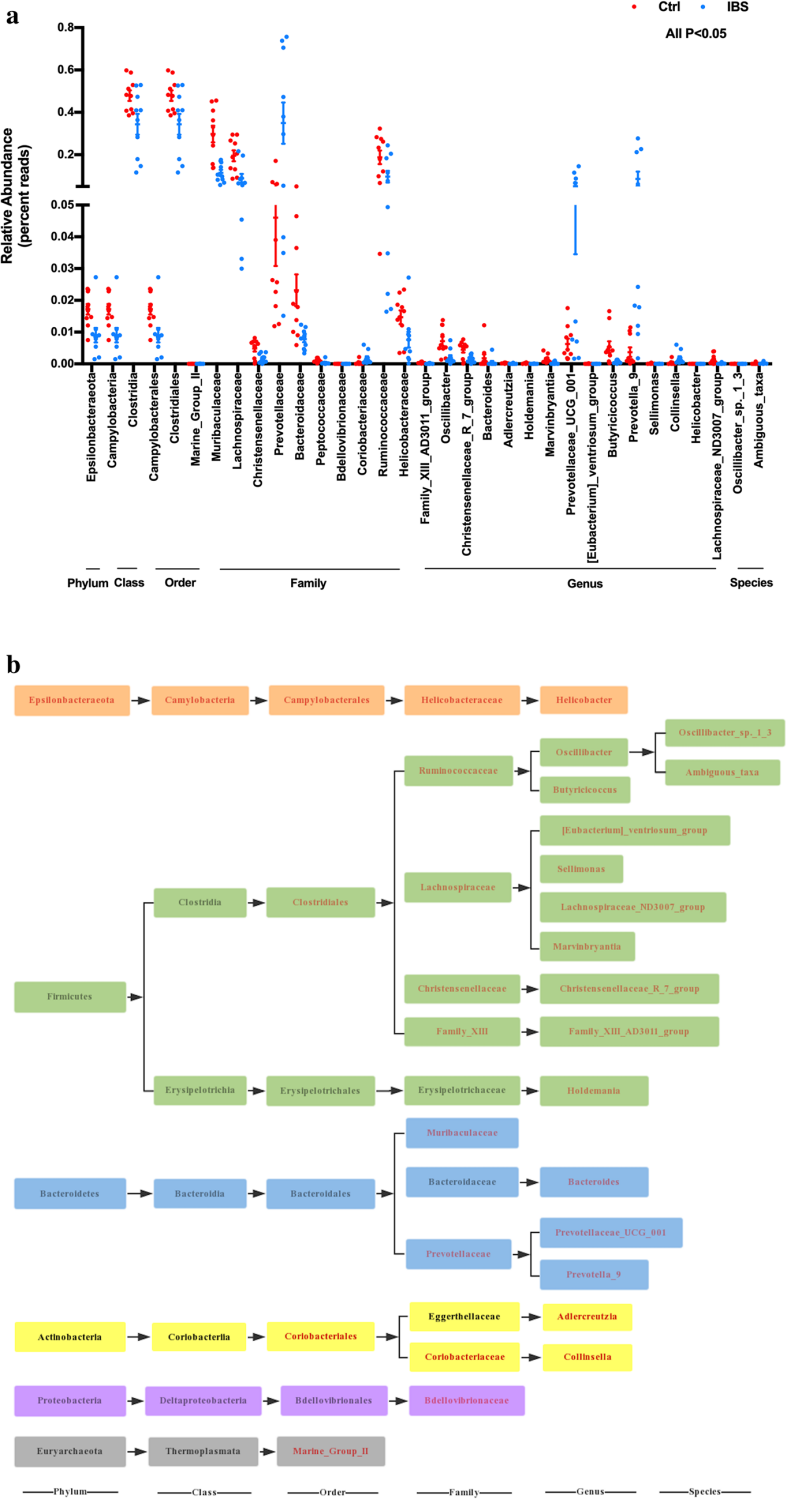
(**a**) The relative abundances in bacterial phylum, class, order, family, genus and species levels in the control (n = 10) and IBS (n = 10) groups, P < 0.05. (**b**) The dendrogram of the different gut microbiome obtained from the control (n = 10) and IBS (n = 10) groups, the statistical difference is presented in red (*P* < 0.05). Statistical significance was determined by one-way analysis of variance (ANOVA) with Fisher’s least significant difference (LSD) post hoc test.

The taxonomical analysis indicated that Firmicutes, Bacteroidetes and Proteobacteria were the major phyla of the gut microbiota in both groups. It is noteworthy that, compared to the control group, the relative abundance of Epsilonbacteraeota was decreased in the IBS group at the phylum level. The 16 s rRNA sequencing indicated that Bacteroides, Helicobacter, Prevotella_9, Prevotellaceae_UCG-001, Oscillibacter and Christensenellaceae_R-7_group were the predominant genera at the genus level in both groups. Furthermore, the microbial composition changes of the ileocecus contents in the IBS group were as follows: Prevotella_9, Collinsella and Prevotellaceae_UCG-001 significantly increased, and Bacteroides, Oscillibacter, Holdemania, Helicobacter, Sellimonas, Butyricicoccus, Lachnospiraceae_ND3007_group, Adlercreutzia, Christensenellaceae_R_7_group, Family_XIII_AD3011_group, and Marvinbryantia significantly decreased (Fig. 3a,b). The ROC curve analysis also revealed that Prevotella_9, Marvinbryantia, Oscillibacter, and Adlercreutzia were the potential biomarkers for separating IBS rats from control rats (Table 1).

**Table 1:** ROC curve analysis between the control and IBS groups.

Name	AUC	Cut off	Sensitivity (%)	Specificity (%)
**Microbe**				
Prevotella_9	0.93	1.121	80	100
Adlercreutzia	0.86	1.40 × 10^−2^	80	90
Marvinbryantia	0.89	7.60 × 10^−2^	80	90
Oscillibacter	0.88	4.28 × 10−1	80	80
**Metabo**				
dl.Phenylalanine	0.85	1.39 × 10^−1^	80	80
dl-3-Phenyllacticacid	0.83	4.00 × 10^−3^	80	80
Nicotinamide	0.86	1.14 × 10^−1^	80	80
1-Naphthol	0.88	3.00 × 10^−3^	80	90

Markers with an area under the ROC curve (AUC) above 0.8 (P < 0.05); both sensitivity and specificity above 80% were selected.

Furthermore, the KEGG pathway analysis revealed that 27 KEGG pathways in level 2 were significantly decreased in the IBS group, when compared to the control group (Supplemental material 2). In order to avoid the impact of abundance on the predicted metagenomes, Bonferroni adjusted *P*-values were performed to analyze the KEGG pathways in level 3 with significantly different abundances. Compared with the control group, one KEGG pathway (Arachidonic acid metabolism, P = 0.007) was significantly enriched in the IBS samples, while two KEGG pathways (Amyotrophic lateral sclerosis, P = 0.016; Penicillin and cephalosporin biosynthesis, P = 0.023) were significantly decreased in the IBS samples. In addition, the high-level phenotypical difference analysis indicated that the abundance of oxidative stress tolerant bacteria was higher, when compare to that in the IBS group (P = 0.035, Supplemental material 3).

### The gut metabolic profiles altered in the IBS model

**Figure 4:**
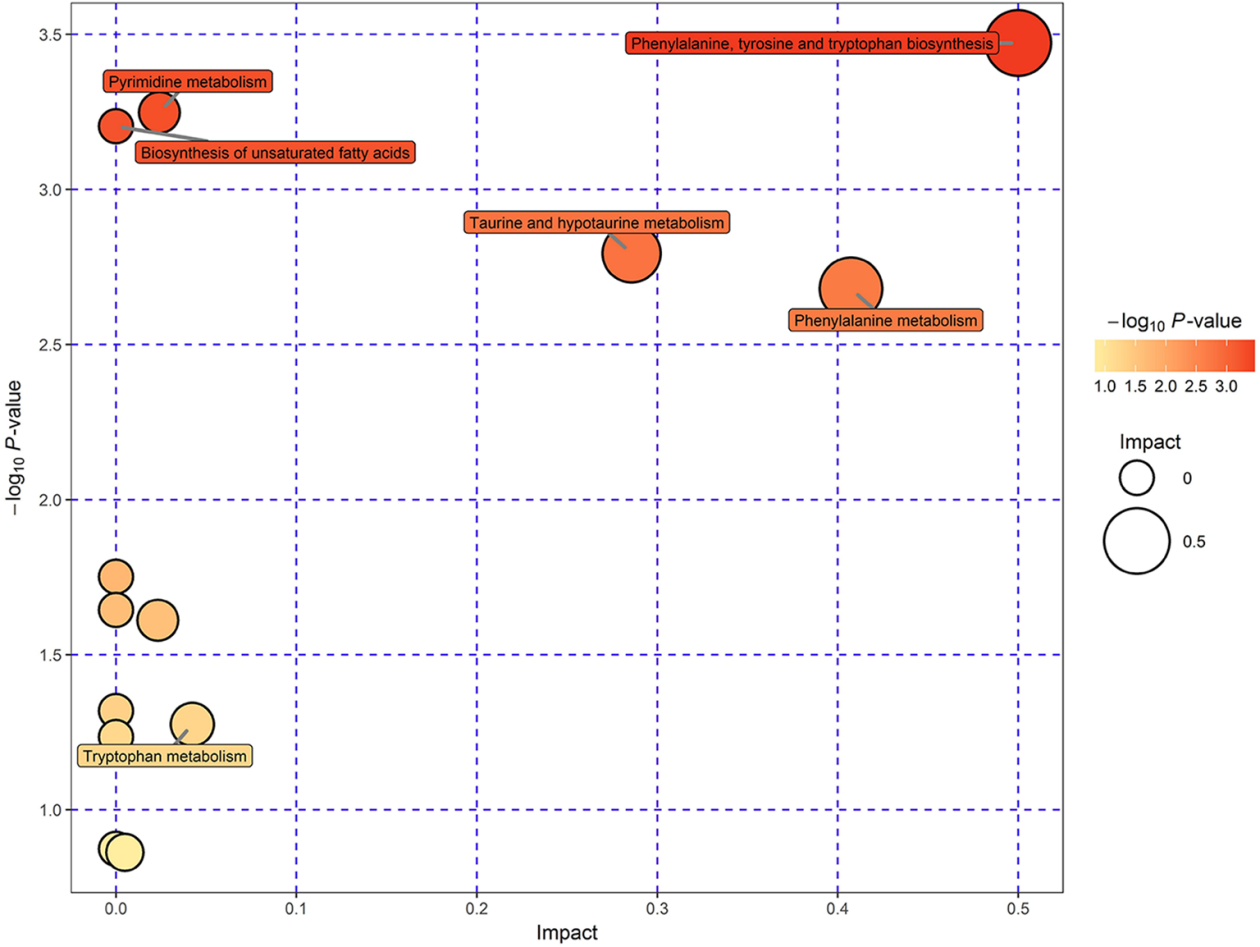
The pathway analysis of metabolites in the control and IBS groups (bubble diagram). Each bubble represents a metabolic pathway. The abscissa and bubble size indicate the size of the influence factor of the pathway in the topological analysis. The larger the size, the more obvious the influence became. The ordinate of the bubble and bubble color indicate the enrichment extent (negative common logarithm, i.e. − log10 P-value). The deeper the bubble color, the more significant the enrichment of metabolites became.

The metabolic profile difference between control and IBS rats was examined using the OPLS-DA model (Supplemental material 4). A total of 24 differentially relatively abundant metabolites with values of variable importance for projection (VIP) larger than 2.0 were identified between control and IBS rats: tryptamine, l-phenylalanine, d-allose, glycyl-l-leucine, deoxycytidine, 2′-deoxyuridine, deoxyguanosine, tyramine, nicotinamide, 1-methylhistamine, 1-naphthol, dl-3-phenyllactic acid, 5-aminopentanoic acid, betaine aldehyde, 4-hydroxycinnamic acid, benzenamine, *N*,*N*-dimethyl, dl-phenylalanine, Val-Ala, inosine, 2′-deoxy, Arg-Thr, .alpha.-CEHC, Ile-Met, Pro-Val, and 1-(9Z-octadecenoyl)-sn-glycero-3-phosphocholine (Table 2). Among these metabolites, five metabolites significantly decreased, while 19 metabolites significantly increased in experimental rats, when compared to normal controls. As indicated by the ROC curve analysis, dl.phenylalanine, dl-3-phenyllactic acid, nicotinamide and 1-naphthol can be used to distinguish IBS rats from controls (Table 1). Furthermore, phenylalanine, tyrosine and tryptophan biosynthesis, pyrimidine metabolism, and the biosynthesis of unsaturated fatty acids were detected to be involved in the most relevant metabolic pathways influenced by IBS, with impact values of 0.500, 0.024 and 0.013, respectively (Fig. 4).

**Table 2:** The different metabolic profiles between the control and IBS groups.

Metabolites	RT(min)	m/z	Formula	VIP	FC	I/C
Tryptamine	175.481	159.0888559	C10H12N2	2.527236799	10.23388048	↑*
l-Phenylalanine	236.869	164.0712088	C6H5CH2CH(NH2)COOH	3.257868165	2.288921906	↑*
d-Allose	279.83	180.0658534	C6H12O6	3.065588271	1.818003479	↑*
Glycyl-l-leucine	262.2775	187.108015	C8H16N2O3	2.614441928	1.846529973	↑*
Deoxycytidine	191.3785	226.0824478	C9H13N3O4	2.874497655	3.294664878	↑*
2′-Deoxyuridine	110.218	227.0664823	C9H12N2O5	2.299523127	2.99132186	↑*
Deoxyguanosine	212.8555	266.0885534	C10H15N5O5	2.467567161	2.92568597	↑*
Tyramine	237.1	120.0820296	C8H11NO	2.525848616	2.097018042	↑*
Nicotinamide	54.07	123.0563525	C6H6N2O	3.099516054	0.309628866	↓*
1-Methylhistamine	329.379	126.103013	C6H11N3	2.444876563	1.970193601	↑*
1-Naphthol	174.7735	127.0550458	C10H8O	3.304590231	2.411010172	↑**
dl-3-Phenyllactic acid	236.99	131.0497754	C9H10O3	2.476377028	1.948938956	↑*
5-Aminopentanoic acid	221.6005	156.0409947	C5H11NO2	2.298235839	0.865085912	↓*
Betaine aldehyde	174.4855	162.1119043	C5H12NO	2.674773238	5.473600476	↑**
4-Hydroxycinnamic acid	279.6805	165.0557845	C9H8O3	2.315158624	1.839011899	↑*
Benzenamine, *N*,*N*-dimethyl	279.919	166.0589864	C8H11N	2.315944227	1.730375377	↑*
dl-Phenylalanine	237.137	166.0879951	C9H11NO2	2.570877535	1.947206392	↑*
Val-Ala	238.291	189.1247185	C8H16N2O3	2.312833141	1.829515885	↑*
Inosine, 2′-deoxy	168.3885	253.0953919	C10H12N4O4	2.394100456	1.859738405	↑*
Arg-Thr	376.981	258.1569804	C10H21N5O4	2.155277835	0.675440202	↓*
.alpha.-CEHC	367.2525	261.1464046	C16H22O4	2.068675287	0.817708426	↓*
Ile-Met	187.6065	323.1622355	C11H22N2O3S	2.28087105	6.810768267	↑*
Pro-Val	222.1	429.2642371	C10H18N2O3	2.224661084	0.425122604	↓*
1-(9Z-Octadecenoyl)-sn-glycero-3-phosphocholine	170.7245	522.3594219	C26H52NO7P	2.176734582	1.895295363	↑*

*RT* retention times, *VIP* variable importance for projection, *I/C* IBS group compared to controls, *FC* fold change.

↑: upregulated, ↓: downregulated; *P < 0.05, **P < 0.01.

### Correlations between the gut microbiome and metabolome

**Figure 5:**
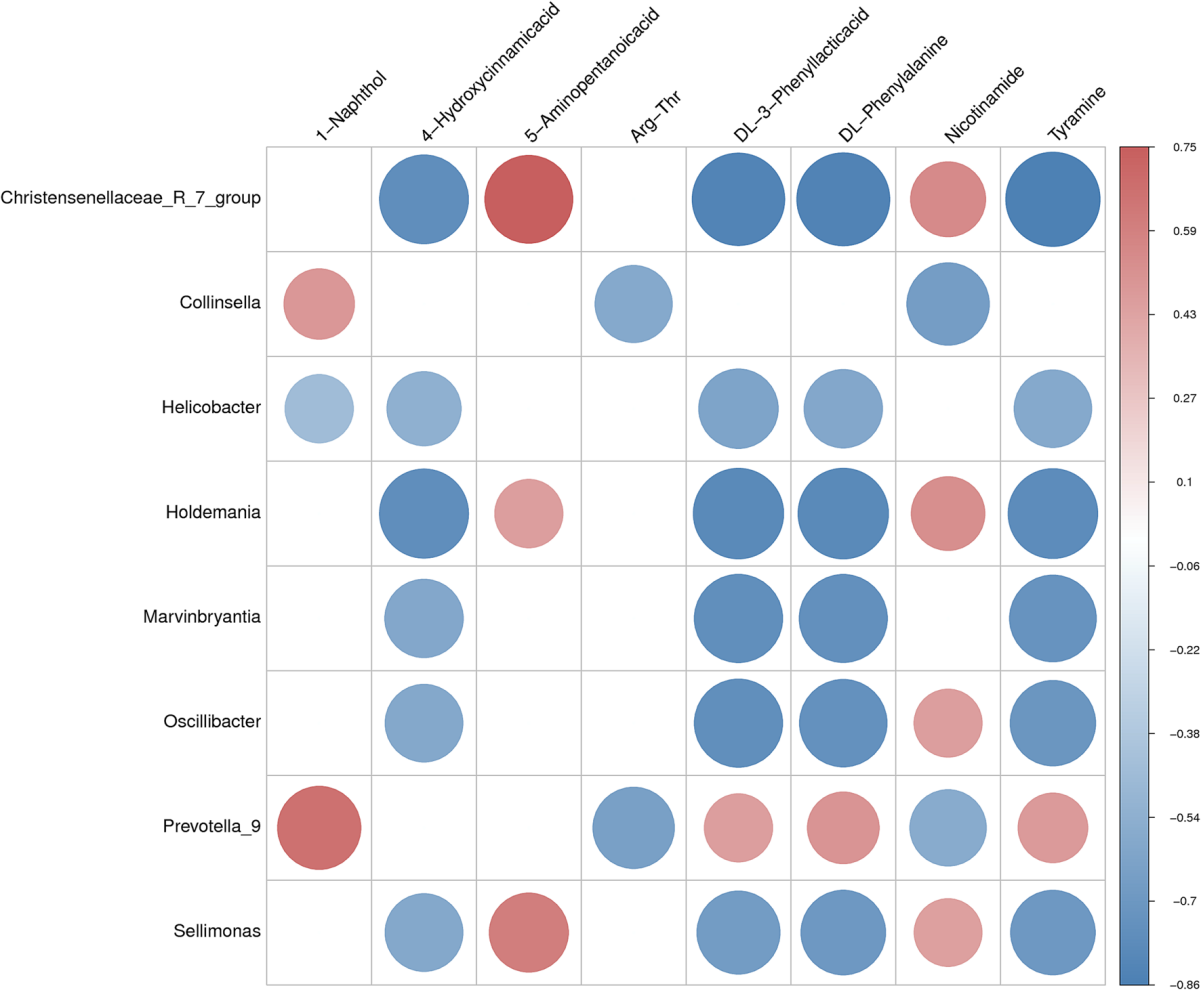
The correlation plot summarizing the functional correlation between the perturbed gut bacteria genus and altered gut metabolites between the control and stress-induced IBS rats.

The Spearman correlation analysis for significantly different metabolites and microbes was performed to obtain the relationships between metabolites and microbes (Fig. 5). As a result, Prevotella_9, which increased by 23.25-fold in IBS rats, was found to be positively correlated with 1-Naphthol, but negatively correlated with Arg-Thr. Similarly, dl-phenylalanine, dl-3-phenyllactic acid and Tyramine were negatively correlated with Sellimonas, Marvinbryantia, Holdemania, Christensenellaceae_R_7_group and Oscillibacter, respectively. In addition, 4-Hydroxycinnamic acid was negatively correlated with Holdemania and Christensenellaceae_R_7_group. Furthermore, 5-Aminopentanoicacid was positively correlated with Sellimonas and Christensenellaceae_R_7_group, while Nicotinamide was negatively correlated with Collinsella. In summary, stress can induce a significant difference in the structure/composition of the gut microbiome, and substantially alter the metabolomic profile.

## Discussion

The present study revealed the significant differences in gut microbial community abundance in the levels of phylum, class, order, family, genera and species between IBS and control rats. The metabolic profile was changed in stress-induced IBS rats. In addition, the altered gut microbiota had significant correlations with metabolites. These findings may provide mechanistic insights on IBS pathophysiology in the aspects of gut microbiome and metabolome. Furthermore, these may provide new potential biomarkers and therapeutic targets for the treatment of IBS disease, and preventing its progression. A decrease in microbial diversity in the present IBS model was observed through the 16S rRNA gene sequencing of ileocecum contents, indicating that the microbial diversity was affected by stress treatment. Interestingly, microbial diversity was also reported to be decreased in the noxious-stress animal model^29^ and IBS patients^30,31^, suggesting that gut microbial diversity is important for preventing IBS development. Furthermore, it was also reported that the number of oxidative stress tolerant bacteria increased in the stress-induced IBS rat model, suggesting that the gut microbiota can modify itself to accommodate stress.

The abundance of certain key taxa and microbial clades substantially differed between the IBS and control groups. At the genus level, Prevotella_9 and Collinsella were significantly over-represented in the stress-induced IBS samples, while Christensenellaceae_R-7_group, Bacteroides, Marvinbryantia, Butyricicoccus, Adlercreutzia, Oscillibacter and Lachnospiraceae_ND3007_group were under-represented. The overabundance of Prevotella in the IBS model was consistent with the results reported by previous studies^7,32–34^. The Prevotella may be involved in inducing visceral hypersensitivity and exacerbating the symptoms of IBS by promoting carbohydrate fermentation and impairing intestinal mucosal immune function^35,36^. Prevotella overabundance was also correlated to other systemic conditions with high-fat diet, such as obesity^37^ and non-alcoholic fatty liver disease^38^. Similarly, the 5.5-fold increase in abundance of the genus Collinsella in IBS rats may have the potential to play a significant role in intestinal gas production, and have a proinflammatory effect, thereby contributing to IBS development^39^. In addition, the decrease in butyrate-producing bacteria^40–43^ in the IBS model, such as Christensenellaceae_R-7_group^44^, Bacteroides^45^, Butyricicoccus^46^, Adlercreutzia^47^, Marvinbryantia^48^, Oscillibacter^7^ and Lachnospiraceae_ND3007_group^49^, was also reported to be variously associated with IBS in humans, as well as in rat models of visceral pain. Butyrate has been proposed to play a key role in maintaining gut homeostasis and epithelial integrity, since it serves as the main energy source for colonocytes. This can directly inhibit histone deacetylases, and interfere with proinflammatory signals^50,51^. The enrichment of arachidonic acid metabolism was also observed in the IBS group, which is involved in a classical inflammation signal pathway^52^. In addition, a recent study conducted by Aguilera-Lizarraga et al.^53^ on the microbiota of IBS patients with meal-induced abdominal pain also support the notion that inflammation may contribute to the initiation of IBS visceral hypersensitivity, since pro-inflammation-related bacteria were significantly elevated in the IBS groups (*Staphylococcus aureus* and *Streptococcus pyogenes*).

The enrichment of this pathway in the IBS group may contribute to the development of IBS. The metabolic potential of gut microbes has been shown to modulate the health status of the host^11,54^. Alterations in the relative abundance of 10 major metabolites were observed, in which three of these (Nicotinamide, Pro.val and Arg.Thr) significantly decreased in the IBS samples, while seven of these (4-Hydroxycinnamic acid, l.Phenylalanine, dl.Phenylalanine, dl-3-Phenyllactic acid, Tryptamine, Tyramine and 1-Naphthol) significantly increased. Nicotinamide is highly concentrated in healthy colons, and can reduce inflammation and carcinogenesis via affecting T-cell differentiation^55–57^. The other two decreased metabolites, Arg.Thr^58^ and Pro.val^59^, can participate in the synthesis and decomposition of essential or non-essential amino acids, and contribute to the maintenance of immune stability. The decreased abundance of these three metabolites in the stress-induced IBS rat model indicates that colonic immune instability might be affected. The increased metabolic of dl-3-Phenyllactic acid^60^ and 4-Hydroxycinnamic acid^61,62^, which has many pharmacological properties including immune-modulatory, anti-inflammatory and antioxidant effects, may be involved in regulating the imbalance of intestinal immunity. These two isoforms of Phenylalanine, l.Phenylalanine and dl.Phenylalanine have similar physiological efficacy, and these were detected to be highly produced in the stress-induced model, indicating the low bioavailability of metabolites in the intestine of stress rats. These two metabolites were reported to be associated with inflammation^63^, and were also reported in a previous IBS-related study^64^. Furthermore, the increased level of Tryptamine and Tyramine was been considered to be involved in contributing to the hypersensitivity in IBS and antioxygenation^65,66^. In a recent study, tryptamine was significantly elevated in stool samples obtained from IBS-D patients, and this was confirmed to stimulate colonic epithelium fluid secretion^67^. The 1-Naphthol is considered as the product of benzene and nepthalene biotransformation, which has a strong antioxygenation effect^68^. More importantly, l.Phenylalanine, dl.Phenylalanine, Tryptamine and Tyramine are essential aromatic amino acids, which are the major nutrient and closely linked to host energy metabolism. The enrichment of these metabolites indicate that the energy metabolism was perturbed by stress. Consequently, the metabolic changes induced by stress in IBS rats might be closely associated with the imbalance of the colonic immune system, intestinal hypersensitivity, increased energy metabolism and adaptive antioxidant capacity.

The changes in gut microbiota and metabolic phenotypes have been widely used to understand the mechanism of disease development^69,70^. In the present study, the significantly different microbes and metabolites between the IBS and control groups were examined, and the metabolite-microbial relationship was evaluated. Eight metabolites were correlated with at least one of eight bacteria after the correlation analyses. Tyramine, l.Phenylalanine, and dl-3-Phenyllacticacid increased in the IBS group, and were negatively correlated with five butyrate-producing bacteria. Among these, Prevotella_9 and Collinsella were the positively-related bacteria, while the remaining six bacteria (Christensenellaceae_R_7_group, Marvinbryantia, Holdemania, Sellimonas, Oscillibacter, and Helicobacter) were negatively correlated with the metabolites. The 1-Naphthol had a strong correlation with Prevotella_9, which might suggest that the capability of oxidative stress tolerance was enhanced in IBS rats. The anti-inflammatory metabolite Nicotinamide was closely and negatively correlated with Collinsella, suggesting that Nicotinamide production can be inhibited by Collinsella. Thus, these correlation differences may play a role in discovering the relationship between IBS and gut microenvironment variation in the stress model.

In summary, the present study combined 16S rRNA gene sequencing and metabolomics methods to investigate the gut microbiome and metabolic profiles of IBS. The sequencing data revealed that the gut microflora composition was significantly altered in the IBS group, while the metabolomics revealed that a number of metabolites involved in diverse metabolic pathways were perturbed by stress. In addition, the correlation analysis identified that there was an obvious correlation between gut bacteria and metabolites.

## Supplementary Information

Supplementary Information 1.

Supplementary Information 2.

Supplementary Information 3.

Supplementary Information 4.

Supplementary Information 5.

## Data Availability

*Accession codes* The sequence data have been deposited in the SRA database under accession code PRJNA594853.
