# Supplementary figures and images for: Integrative analysis of the gut microbiome and metabolome in a rat model with stress induced irritable bowel syndrome
Source: Sci Rep. 2021 Sep 2;11:17596. doi: 10.1038/s41598-021-97083-z (PMC8413334; doi:10.1038/s41598-021-97083-z)

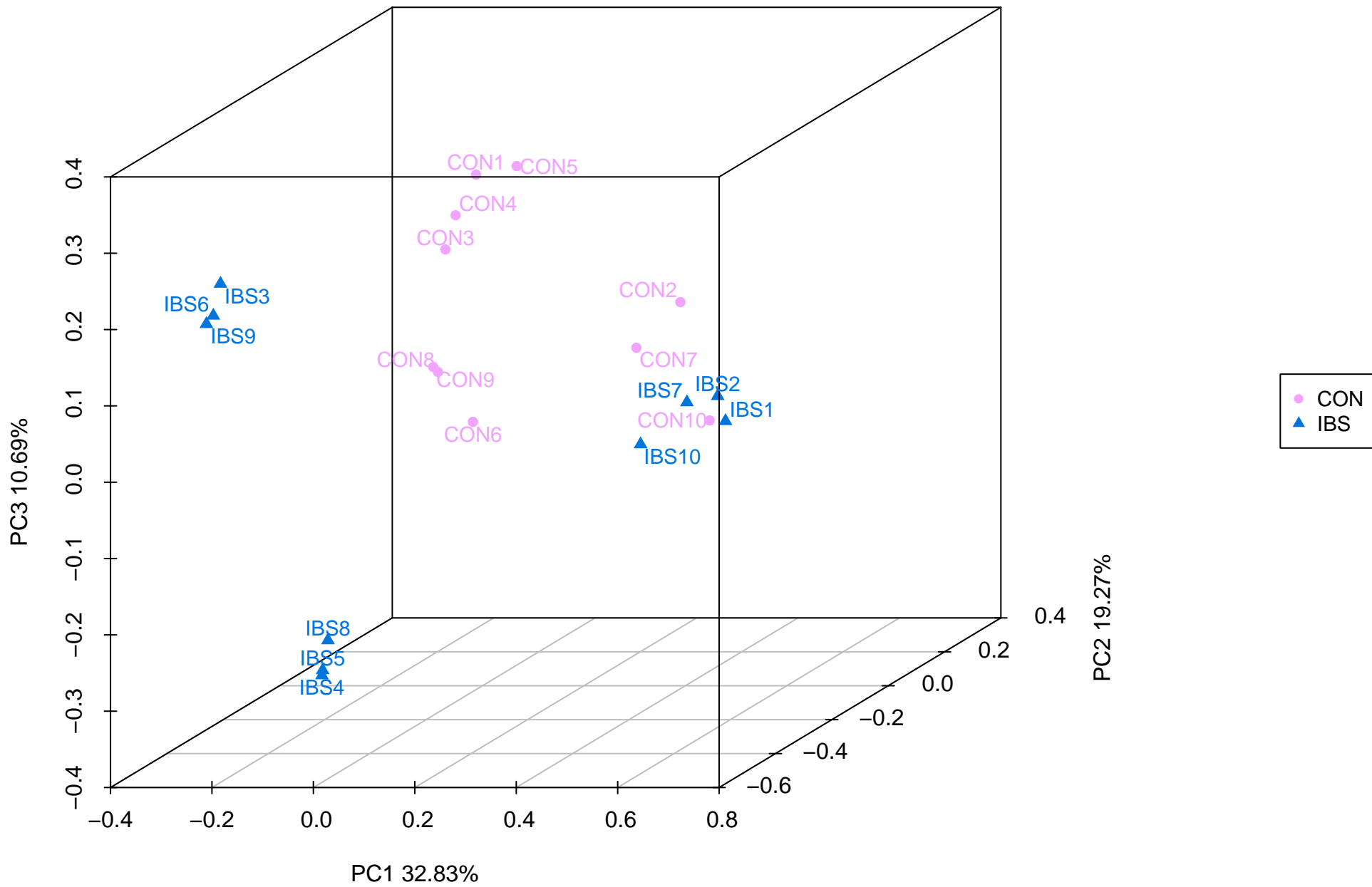

Supplement: Supplementary file 1 — Supplementary Information 1. [file 41598_2021_97083_MOESM1_ESM.zip › Material 1 Principal coordinate analysis (PCoA) of microbiota/PCoA_BrayCurtis_3d.pdf]

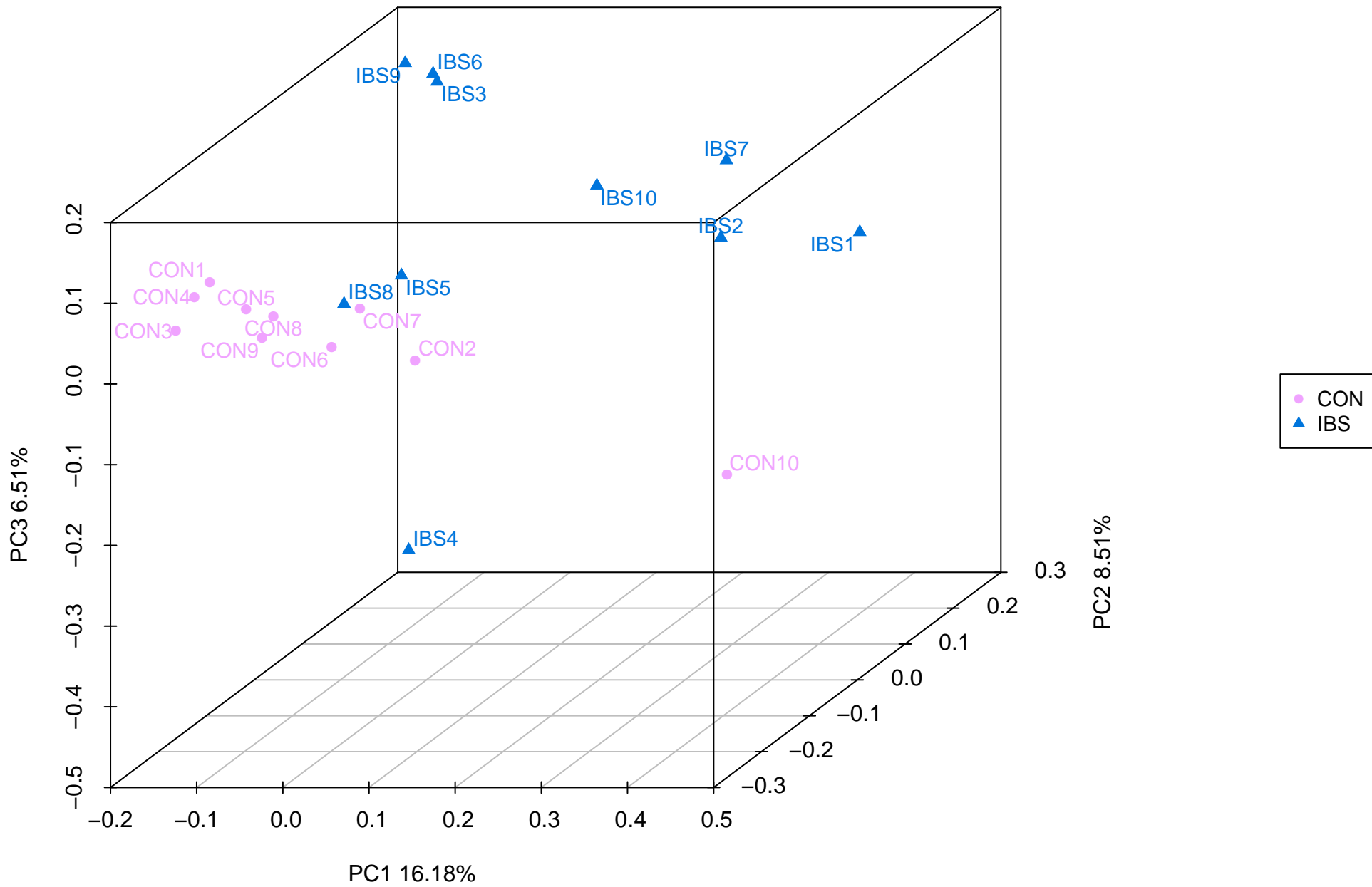

Supplement: Supplementary file 1 — Supplementary Information 1. [file 41598_2021_97083_MOESM1_ESM.zip › Material 1 Principal coordinate analysis (PCoA) of microbiota/PCoA_BinaryJaccard_3d.pdf]

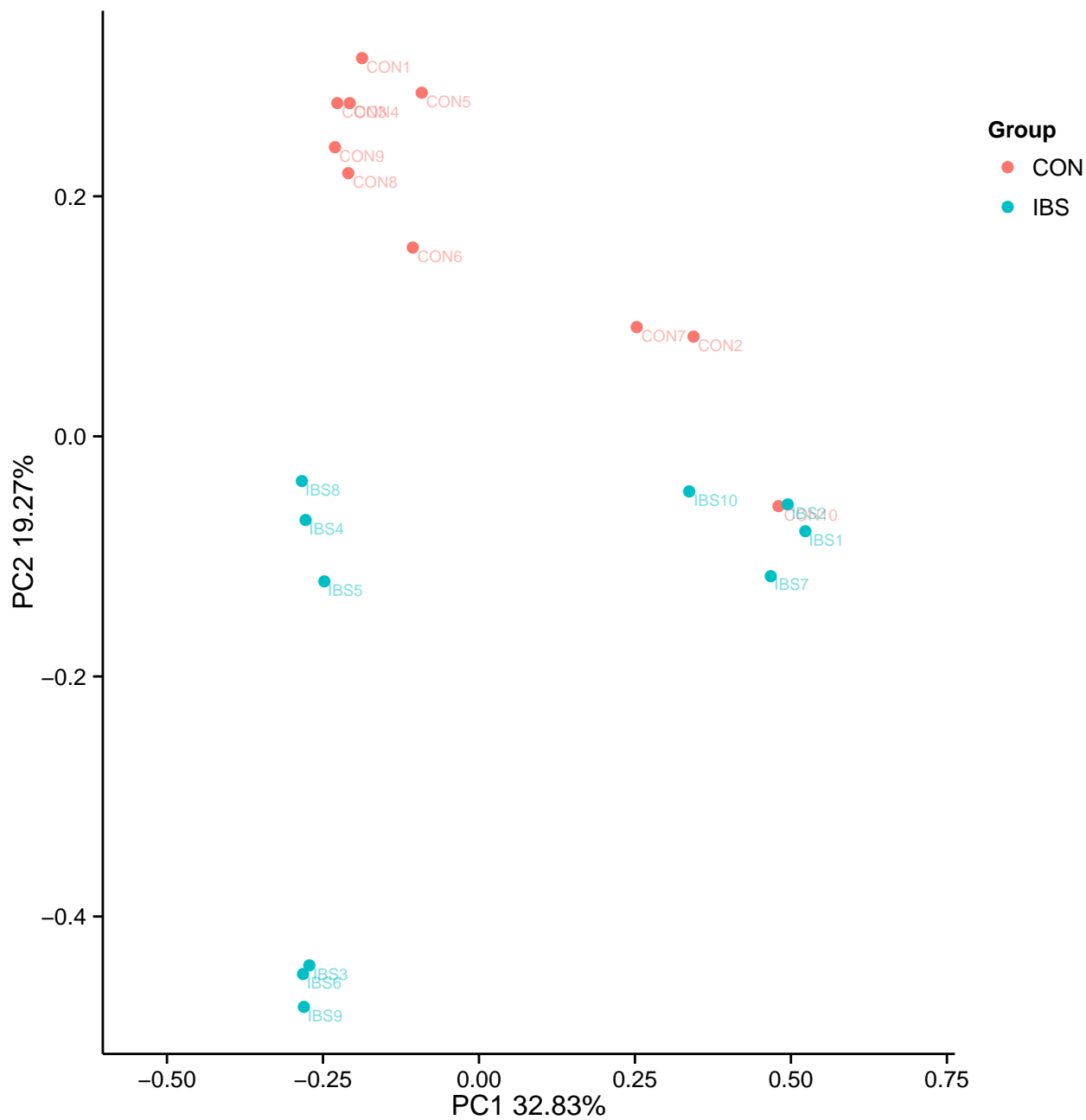

Supplement: Supplementary file 1 — Supplementary Information 1. [file 41598_2021_97083_MOESM1_ESM.zip › Material 1 Principal coordinate analysis (PCoA) of microbiota/PCoA_BrayCurtis_2d.pdf]

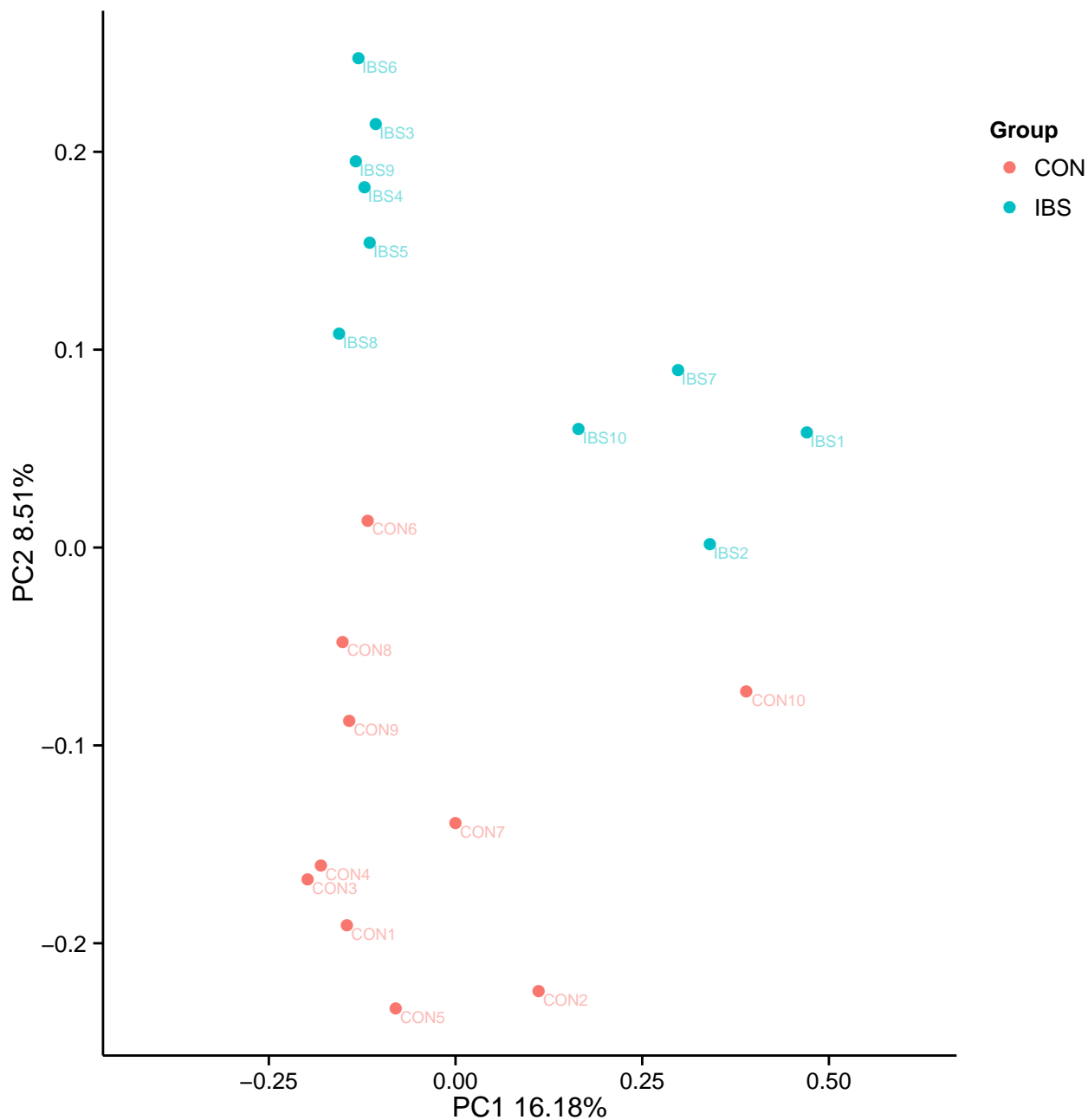

Supplement: Supplementary file 1 — Supplementary Information 1. [file 41598_2021_97083_MOESM1_ESM.zip › Material 1 Principal coordinate analysis (PCoA) of microbiota/PCoA_BinaryJaccard_2d.pdf]

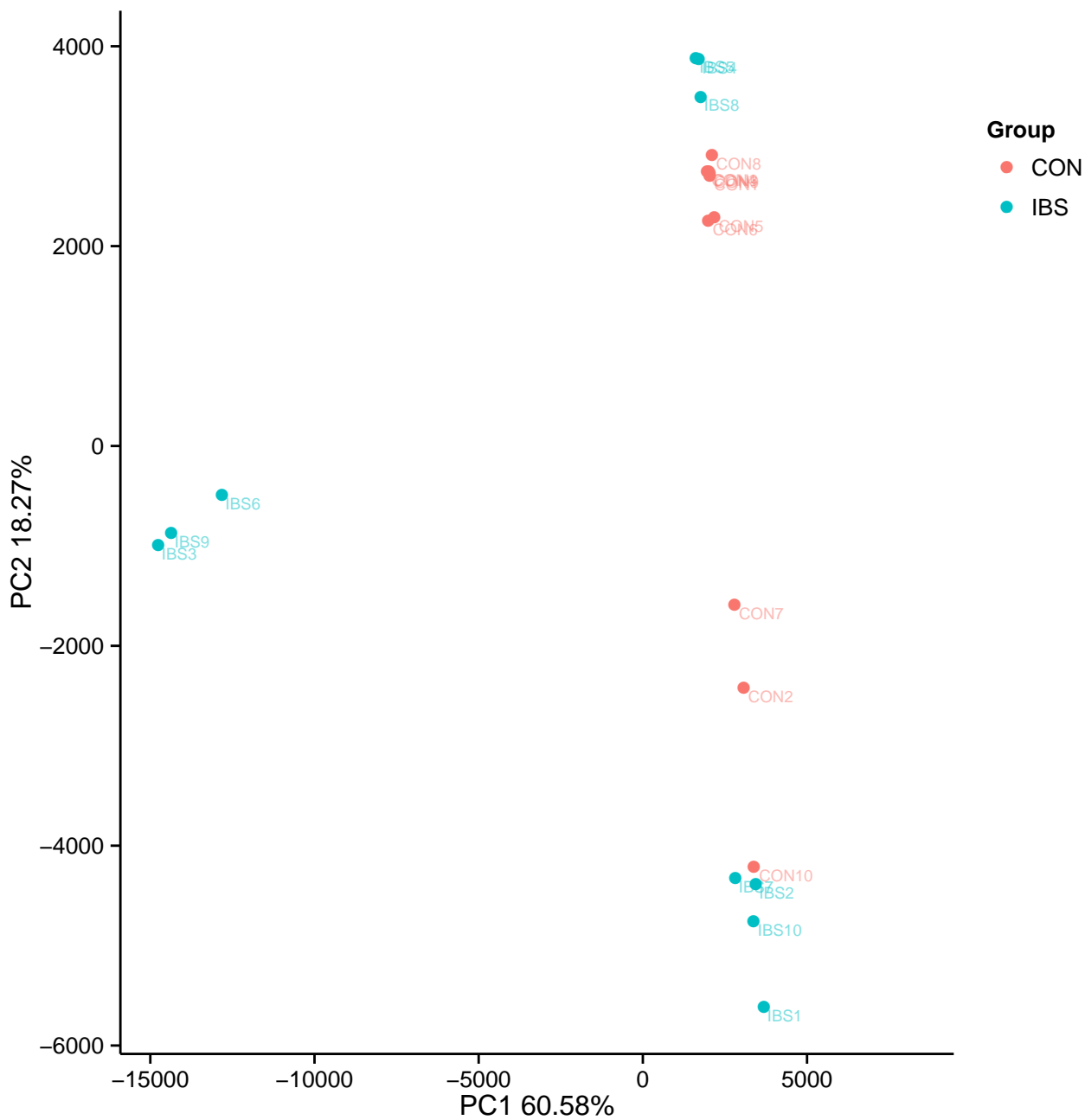

Supplement: Supplementary file 1 — Supplementary Information 1. [file 41598_2021_97083_MOESM1_ESM.zip › Material 1 Principal coordinate analysis (PCoA) of microbiota/PCoA_euclidean_2d.pdf]

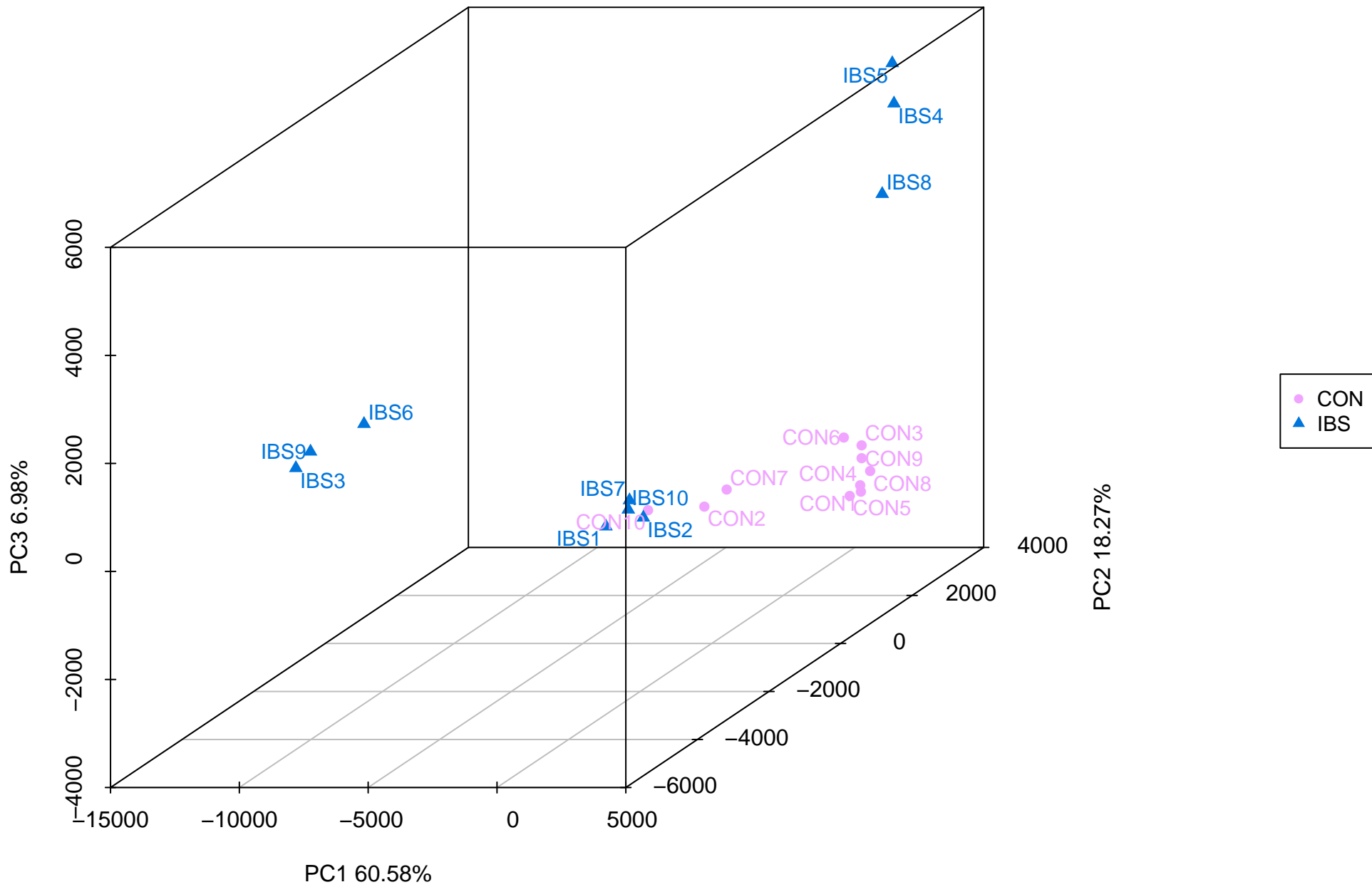

Supplement: Supplementary file 1 — Supplementary Information 1. [file 41598_2021_97083_MOESM1_ESM.zip › Material 1 Principal coordinate analysis (PCoA) of microbiota/PCoA_euclidean_3d.pdf]

Ctrl IBS

All P<0.05

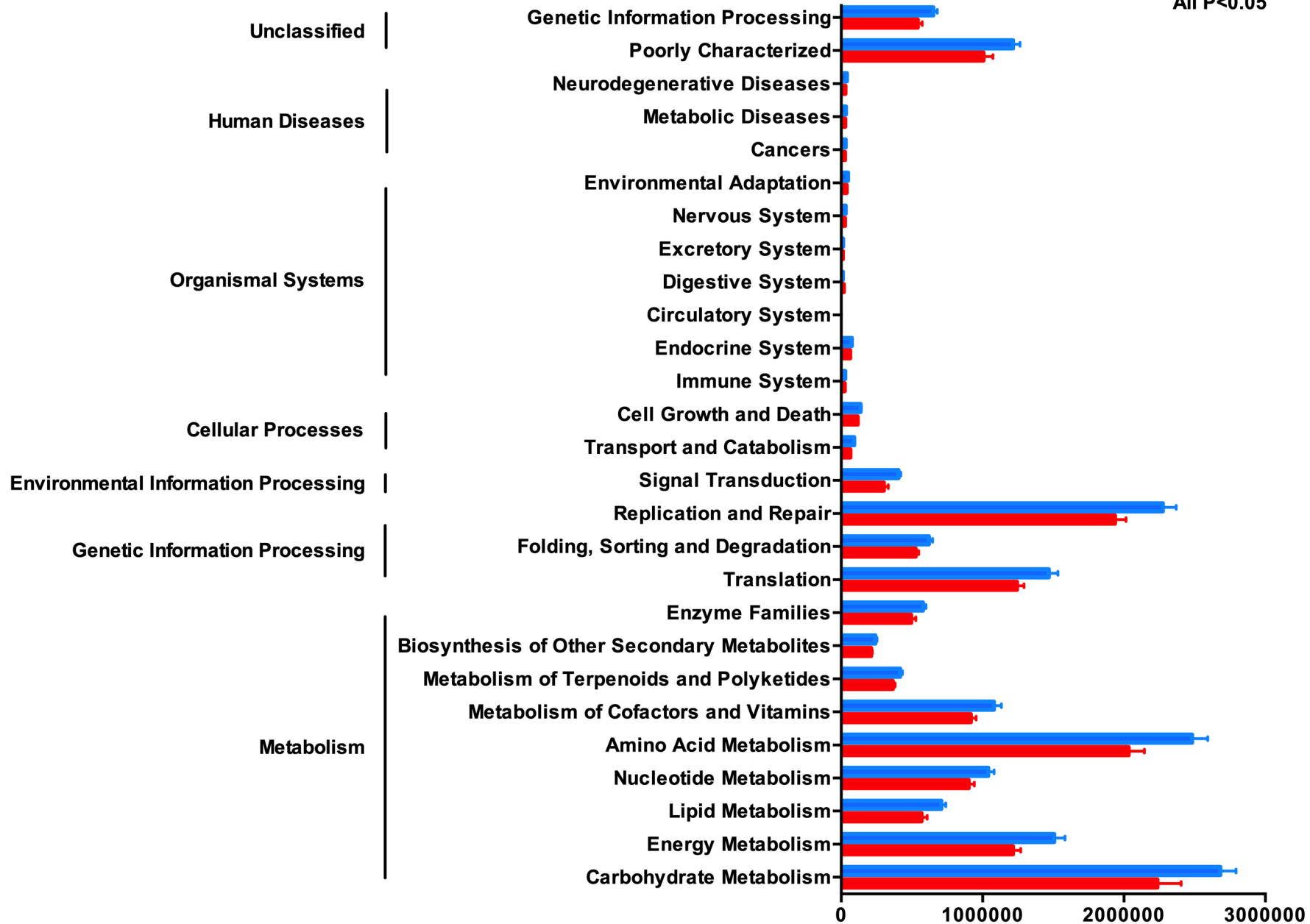

KEGG\_Pathway level 1

KEGG\_Pathway level 2

Absolute abundance of metagenomes

Supplement: Supplementary file 2 — Supplementary Information 2. [file 41598_2021_97083_MOESM2_ESM.pdf]

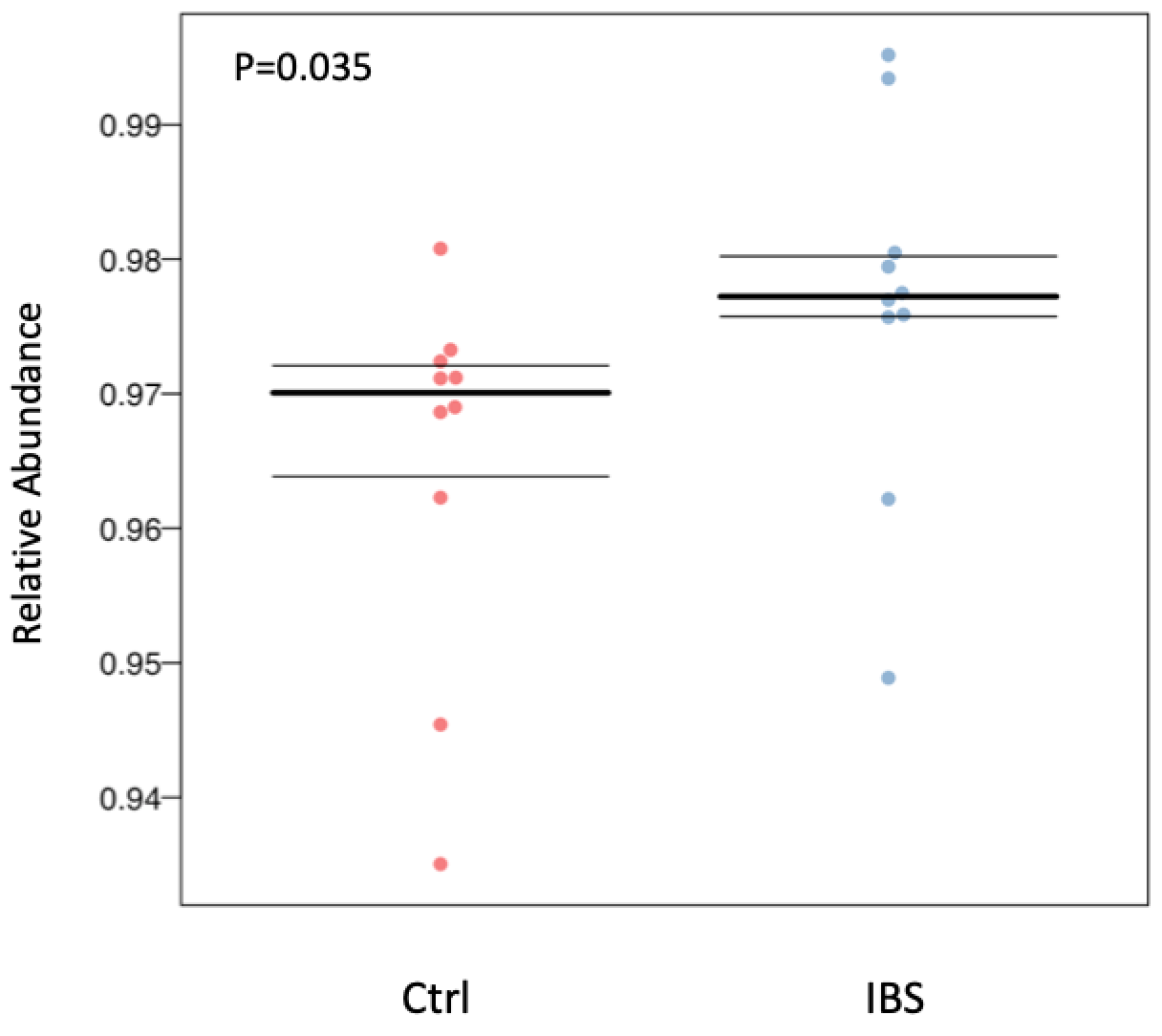

Supplement: Supplementary file 3 — Supplementary Information 3. [file 41598_2021_97083_MOESM3_ESM.png]

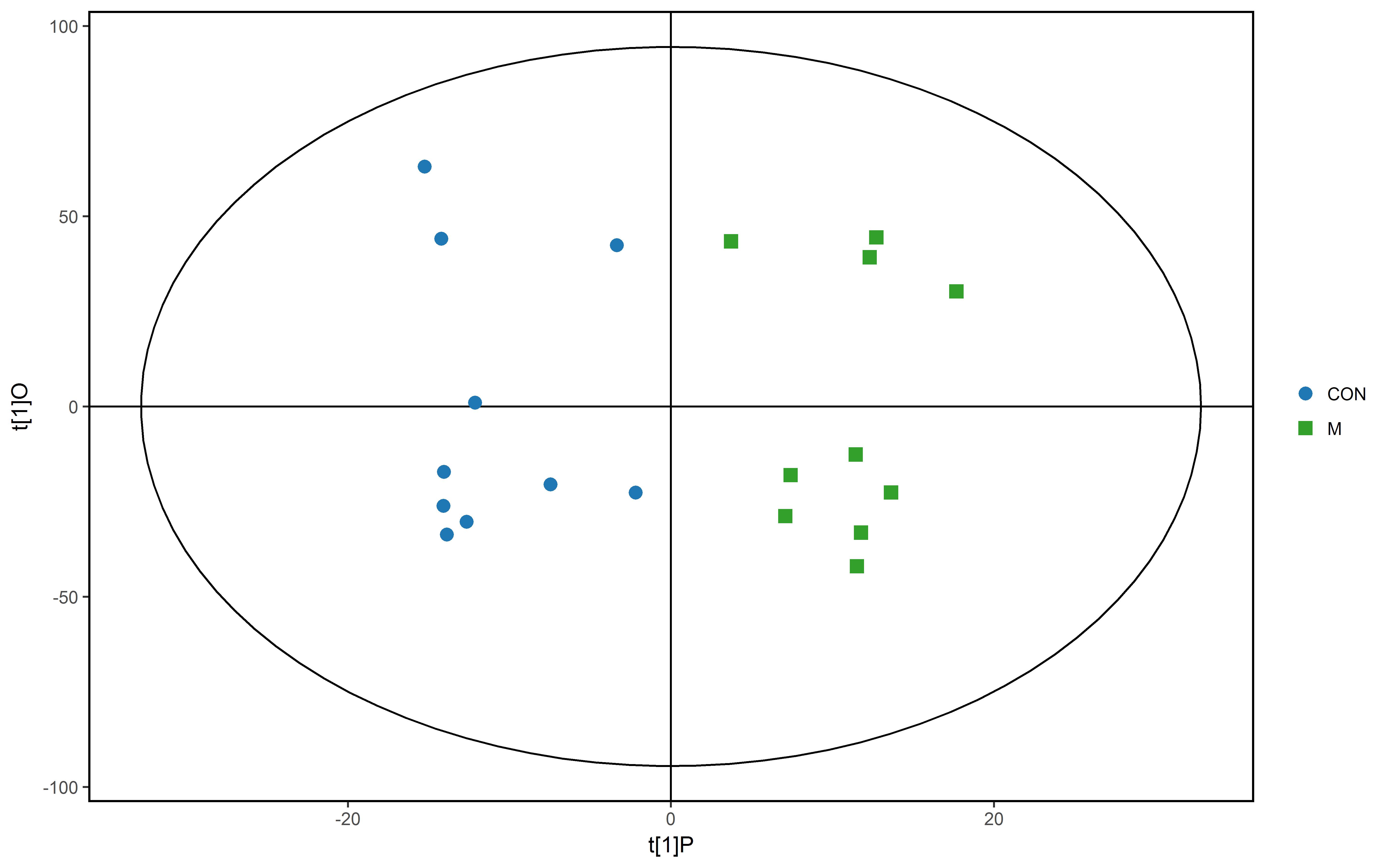

Supplement: Supplementary file 4 — Supplementary Information 4. [file 41598_2021_97083_MOESM4_ESM.jpg]

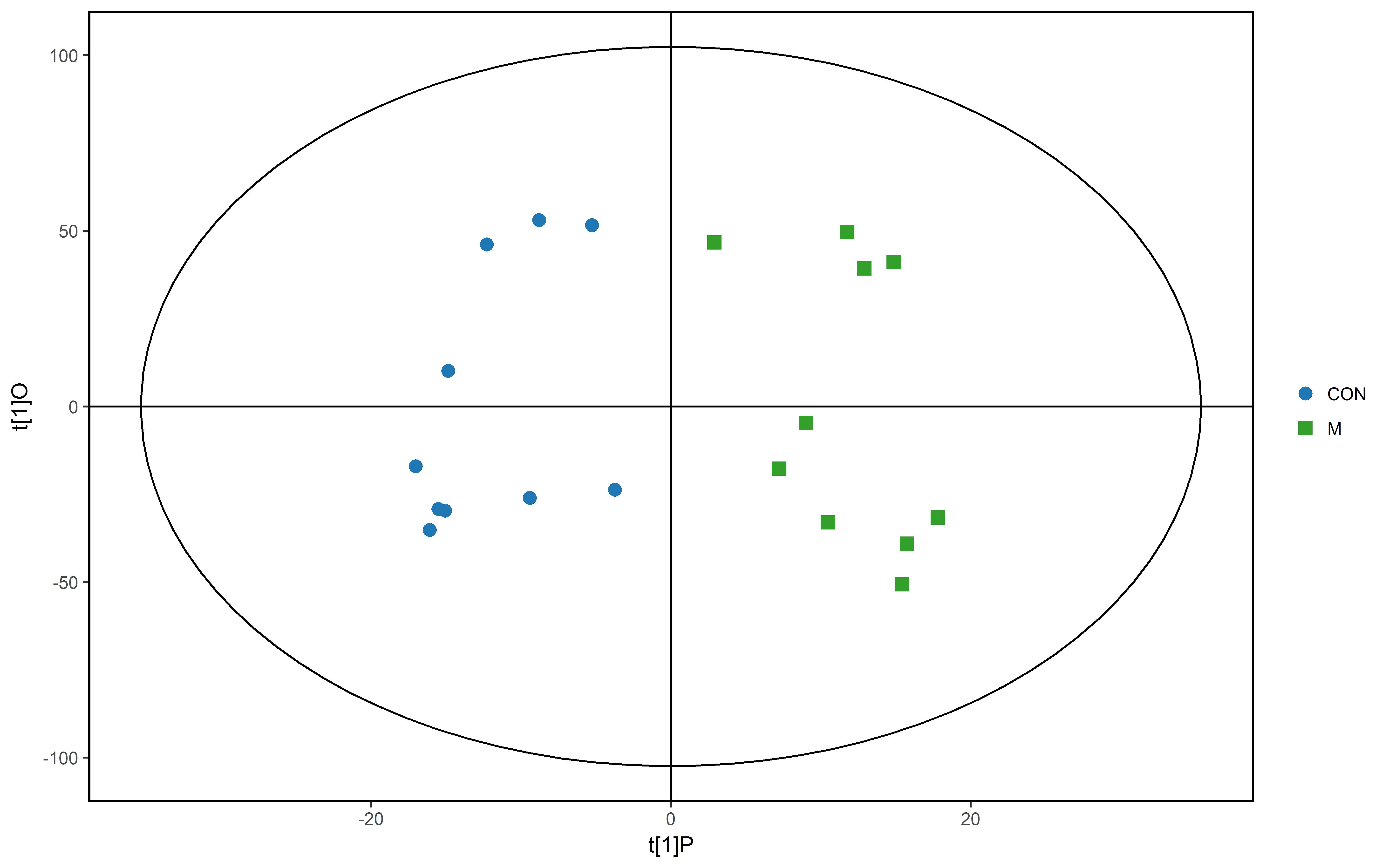

Supplement: Supplementary file 5 — Supplementary Information 5. [file 41598_2021_97083_MOESM5_ESM.jpg]
